# Proteomics profiling reveals lipid metabolism abnormalities during oogenesis in unexplained recurrent pregnancy loss

**DOI:** 10.3389/fimmu.2024.1397633

**Published:** 2024-08-08

**Authors:** Kun Liu, Xiaojuan Xu, Liang Sun, Hongxing Li, Yi Jin, Xiaoling Ma, Bairong Shen, Cesar Martin

**Affiliations:** ^1^ Reproductive Medicine Center, The First Hospital of Lanzhou University, Lanzhou, Gansu, China; ^2^ Biochemistry and Molecular Biology Department of University of the Basque Country (UPV/EHU), Leioa, Spain; ^3^ Institutes for Systems Genetics, West China Hospital Sichuan University, Chengdu, China; ^4^ Department of Molecular Biophysics, Biofisika Institute (UPV/EHU, CSIC), Leioa, Spain

**Keywords:** dDIA, recurrent pregnancy loss, human follicular fluid, apolipoprotein, biomarkers, oogenesis

## Abstract

**Background:**

Unexplained recurrent pregnancy loss (URPL) is a clinical dilemma in reproductive fields. Its diagnosis is mainly exclusionary after extensive clinical examination, and some of the patients may still face the risk of miscarriage.

**Methods:**

We analyzed follicular fluid (FF) from *in vitro* fertilization (IVF) in eight patients with URPL without endocrine abnormalities or verifiable causes of abortion and eight secondary infertility controls with no history of pregnancy loss who had experienced at least one normal pregnancy and delivery by direct data-independent acquisition (dDIA) quantitative proteomics to identify differentially expressed proteins (DEPs). In this study, bioinformatics analysis was performed using online software including g:profiler, String, and ToppGene. Cytoscape was used to construct the protein–protein interaction (PPI) network, and ELISA was used for validation.

**Results:**

Gene Ontology (GO) and Kyoto Encyclopedia of Genes and Genomes (KEGG) enrichment analysis showed that the DEPs are involved in the biological processes (BP) of complement and coagulation cascades. Apolipoproteins (APOs) are key proteins in the PPI network. ELISA confirmed that APOB was low-expressed in both the FF and peripheral blood of URPL patients.

**Conclusion:**

Dysregulation of the immune network intersecting coagulation and inflammatory response is an essential feature of URPL, and this disequilibrium exists as early as the oogenesis stage. Therefore, earlier intervention is necessary to prevent the development of URPL. Moreover, aberrant lipoprotein regulation appears to be a key factor contributing to URPL. The mechanism by which these factors are involved in the complement and coagulation cascade pathways remains to be further investigated, which also provides new candidate targets for URPL treatment.

## Background

Recurrent pregnancy loss (RPL) is one of the most challenging clinical problems in reproductive medicine, with complex and highly heterogeneous etiology, and roughly 50% of them are still unexplained (URPL) (1). Internationally accepted diagnostic criteria for URPL are lacking, and its diagnosis largely relies on eliminatory methods, that is, after a comprehensive screening to exclude possible associated etiologies, including anatomic, genetic, infection, hormonal, immunology, psychological, and environmental factors. As such, it is imperative to understand the underlying mechanisms of miscarriage in order to gain a clear picture of its pathogenesis. Exploring protein profile changes in URPL through mass spectrometry (MS) may shed light on phenotypic-level alterations, unveiling potential pathological mechanisms. This exploration plays a pivotal role in the discovery of novel disease-related biomarkers, facilitating precise diagnosis, prediction, and treatment strategies. As the closest natural environment for egg development, follicular fluid (FF) contains a vast dynamically changing protein network (2) that regulates its growth and development and ultimately impacts fertilization and embryonic potential. Understanding proteomic expression in FF thus provides a better insight into oocyte and follicle maturation and somatic-germ cell communication and brings new insights for the diagnosis, treatment, and prevention of reproduction-related diseases.

To our knowledge, very few studies are available to characterize URPL at the proteomic level. Early protein assays such as two-dimensional gel electrophoresis (2-DE) and two-dimensional fluorescence difference gel electrophoresis (DIGE) were complex to perform and yielded relatively limited protein information. In contrast, MS-based proteomics provides a systematic analysis of the structure, function, and their interactions of abundant and complex proteins expressed in cells. It is also an indispensable technique for interpreting the gene-coding information (3). Tandem mass tag (TMT) and isobaric tag for relative absolute quantitation (iTRAQ) are types of daughter ion-labeled quantitative MS technique that are among the two most widely used MS techniques reported in the literature. Pan et al. (4) employed iTRAQ to analyze four placental villous tissues from patients with URPL and four from normal pregnant women. They identified 314 differentially expressed proteins (DEPs), and network analysis showed that angiotensinogen (AGT), MAPK14, and prothrombin are core factors in early embryonic development. The same interaction network was found by Xiong et al. (5) using TMT in the decidua of patients with early URPL. Another study found 456 DEPs in decidua using iTRAQ and revealed that mitochondrial oxidative stress dysfunction might play an important role in promoting the pathological process of URPL (6). Data-independent acquisition (DIA) is a non-labeled quantitative MS technique that has gained much attention in recent years. Compared with traditional label-free quantitation (LFQ) proteomics, which has no sample number limitation and requires a lower sample protein amount (7), its greatest advantage lies in the efficient determination of relatively low-abundance protein molecules in complex samples (8), greatly improving the confidence of quantitative analysis. Second, DIA has greatly innovated and optimized the MS data acquisition mode and quantitative analysis algorithms. The data-dependent acquisition (DDA) mode used in the LFQ and TMT/iTRAQ relies on primary MS signal quantification, which is restrictive and random in the selection of peptide ions, with more missing data information (9). The DIA mode is for secondary MS signal quantification, with the goal of obtaining complete data to achieve in-depth coverage and precise quantification of proteins. Direct DIA, or DDA-free DIA (dDIA), compared with the traditional DIA analysis strategy, does not need to carry out DDA hierarchical library construction (10). Instead, it uses machine deep learning to directly search the DIA original spectra file to generate libraries. Deep learning scoring is used to find peak fragmentation patterns, predict retention times, and remove false-positive results (11). As a result, the data collection is completer and more comprehensive, with lower randomness and better quantitative accuracy and reproducibility (12, 13).

Therefore, we employed dDIA technology integrated with liquid chromatography–tandem MS (LC-MS/MS) analysis to identify protein expression in FF from URPL patients and patients with infertility secondary to tubal factors. The obtained results may enable us to unravel the characteristic biomarkers or pathways involved in the pathogenesis of URPL.

## Materials and methods

### Sample collection

We recruited women undergoing *in vitro* fertilization (IVF) in the Reproductive Medicine Center of the First Hospital of Lanzhou University (Gansu, China). The subjects were composed of two groups: women with URPL (n=28) and a control group (n=28). URPL was defined as women who experienced clinically spontaneous abortion before 20 weeks of gestation with the same partner for two or more times. Exclusion criteria included endocrine abnormalities (such as diabetes, hyperprolactinemia, polycystic ovarian syndrome, and hypothyroidism) and verifiable causes of abortion, such as anatomical abnormalities, chromosomal abnormalities, antiphospholipid syndrome, or infectious factors. The control group comprised women with no history of pregnancy loss who had experienced at least one normal pregnancy and delivery. Follicular fluid (FF) and peripheral blood were collected on the day of oocyte retrieval, and the supernatant was centrifuged and stored at −80°C.

### Experimental design

The pilot study included 56 FF samples from single follicles and 40 peripheral blood samples from each of the 56 patients. In both groups, eight FF samples were used for dDIA proteomics analysis. Additionally, FF and peripheral blood were collected from 20 patients in each group for additional independent validation.

### Protein digestion

The protein concentration and purity of FF were measured by SDS-PAGE and the Bradford method. In total, 100 μg of protein from each original FF was transferred to a new Eppendorf tube, and then, 50 mM Tris solution was added to make the volume up to 100 μL. Proteins were reduced with 0.5 M TCEP (37°C, 60 min) (ThermoScience77720) and alkylated with 1 M iodoacetamide (40 min, room temperature, away from light). Then, a fivefold volume of precooled acetone was added to the mixture and stored at −20°C overnight to precipitate proteins. After centrifugation at 12,000 × g for 20 min at 4°C, the supernatant was discarded, and 1 mL of 90% (v/v) pre-cooled acetone was added, mixed by vortex and centrifuged again. The supernatant was discarded, and this wash step was repeated twice. Next, protein precipitation was air-dried and dissolved in 100 μL 100 mM TEAB buffer. Trypsin (Promega, Madison, WI) was added in a mass ratio of 1:50 (enzyme: protein) for overnight digestion at 37°C. After desalting with C18 columns, the ultimate peptide concentration was quantified using a peptide quantification kit (Pierce™ 23275) and lyophilized.

### Nano-high-performance liquid chromatography–tandem mass spectrometry measurements

The desalted lyophilized peptides were redissolved in solvent A (LC-MS grade water containing 0.1% formic acid), 9 μL was withdrawn, and 1 μL of 10 × iRT peptide (Biognosys AG, Switzerland) was added to it as an internal standard, followed by LC-MS/MS analysis with an on-line nano-spray ion source. The entire system comprised an Ultimate 3000 liquid phase system in tandem with an Orbitrap Exploris™ 480 mass spectrometer (Thermo Fisher Scientific, USA). A total of 2 μL of sample was applied on a reversed-phase column of 75 μm × 50 cm (Monolithic column, High-resolution Ultra C18, 3 microm particle, 300 A pore size, Uritech, China) with a gradient of 25 min at a flowrate of 1.5 µL/min and a column temperature of 60°C. The chromatographic gradient was as follows (**Table 1**) (mobile phase A, 0.1% aqueous formic acid; mobile phase B, 80% acetonitrile containing 0.1% formic acid).

**Table 1:** The gradient of LC-MS/MS analysis.

Time (min)	B (%)
0	5
15	20
20	30
21	50
21.1	90
22.5	90
22.6	1
25	1

### Quality control

After enzymatic purification of each sample, equal amounts of peptides from each sample were mixed into a quality control (QC) peptide sample. A shot was analyzed by the same LC-MS/MS method as the samples at the beginning, middle, and end of the process.

### Quantitative data processing

MS data were captured using the DIA mode. The electrospray voltage was 2.0 kV, and the MS parameters were set as below: (I) MS—scan range, 350–1,200 m/z; resolution, 120,000@m/z 200; AGC target, 300%; and max. injection time, 50 ms; (II) higher-energy collision-induced dissociation (HCD)-MS/MS—resolution, 30,000@m/z 200; AGC target, 200%; and collision energy, 32%; and (III) variable window acquisition, set up with 60 windows and overlapping serial ports, each window overlapped by 1m/z.

### dDIA MS data analysis

The original data were analyzed library-free in Spectronaut 15 (directDIA) (Biognosys AG, Switzerland) using the Biognosys factory settings. The sequence database was UniProt human. The following parameters were used to conduct the database search: fixed modification, carbamidomethyl (cysteine); variable modification, oxidation (methionine). The false discovery rate (FDR) was set at 1% for both parent ion and peptide levels. Differential protein screening criteria were as follows: p-value not greater than 0.05 and fold change not less than 1.5 after Welch's ANOVA test analysis.

### Bioinformatics analysis

GO annotation, KEGG, Reactome (REAC) pathway, and Wiki pathways (WP) enrichment analyses of DEPs were implemented using the g:Profiler online tool (http://biit.cs.ut.ee/gprofiler/) and further data evaluation with OriginPro 2022 learning edition (OriginLab USA). ToppGene (https://toppgene.cchmc.org/enrichment.jsp) link was employed for enriching disease classes. Furthermore, STRING (http://string-db.org) and Cytoscape v3.9.1 (https://cytoscape.org) were used to construct PPI networks and to disclose hub proteins. Drug–Gene Interaction database (DGIdb) was performed to predict the possible targeting therapeutic drugs in URPL.

### Validation of differential protein expression with enzyme-linked immunosorbent assay

For the DEPs validation, FF and peripheral blood samples from 40 subjects were used for the determination of apolipoprotein (APO) B levels. Enzyme-linked immunosorbent assay (ELISA) kits were purchased from Chichao Biotech (Peking, China). ELISA assays were conducted according to the manufacturer's protocol. Each sample was tested in duplicate.

**Figure 1:**
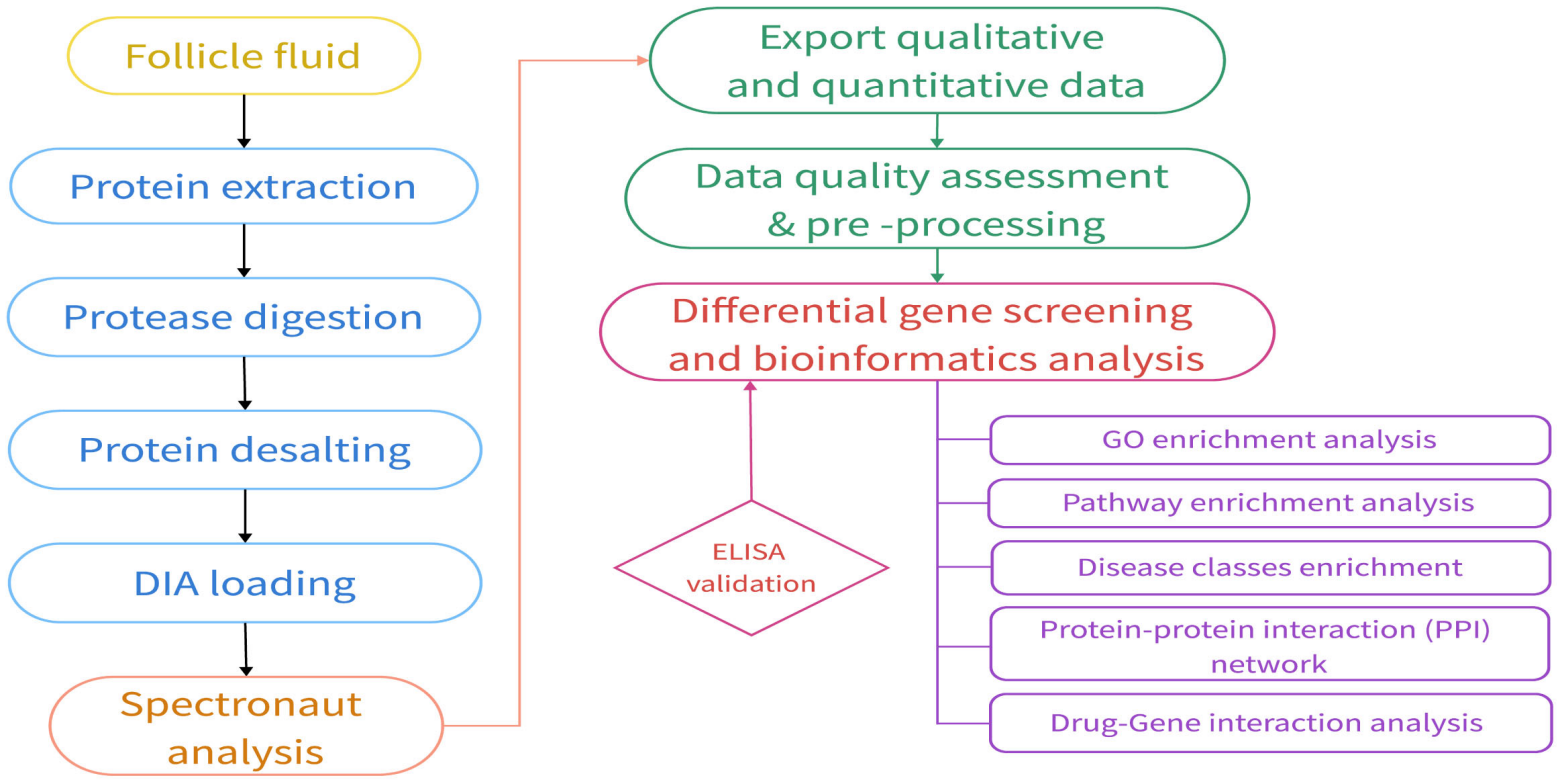
Proteomics experimental and analysis process.

A comprehensive flowchart detailing the experimental design and proteomic analysis is presented in **Figure 1**.

### Statistical analysis

Statistical analysis was performed using SPSS 22 (SPSS Inc.). Basic data were described using means and standard deviation (SD). t-Test or Mann–Whitney U-test was used for group comparisons depending on the circumstance. p-Values below 0.05 were accepted as statistically significant.

## Results

### Essential profiles of participants

A total of 16 FF samples (eight URPL and controls, respectively) were used for dDIA analysis. The main demographic and embryos characteristics are summarized in **Table 2**. No significant differences were found between the two groups. The number of previous miscarriages ranged from 2 to 5 in the URPL group and none in the control group (*p* < 0.0001).

**Table 2:** Comparison of the primary characteristics of patients in the URPL and control groups.

	URPL	Control	*p*-value
Female age	31.50 ± 2.62	32.00 ± 2.14	NS
BMI	21.89 ± 2.76	22.26 ± 3.11	NS
AMH	2.75 ± 2.19	2.97 ± 1.89	NS
E_2_ on oocyte pickup day (pg/mL)*	4139 ± 2212	3327 ± 1231	NS
P on oocyte pickup day (ng/mL)*	1.33 ± 1.12	1.12 ± 0.66	NS
No. of oocytes retrieved	10 ± 3	11 ± 4	NS
No. of 2PN	9 ± 6	10 ± 4	NS
No. of usable embryo on D3	8 ± 4	8 ± 2	NS
No. of previous RSA	3.50 ± 1.07	0	<0.0001

*E_2_, estradiol; P, progesterone.

Data indicate mean ± SD.

### dDIA proteomic profile

**Figure 2:**
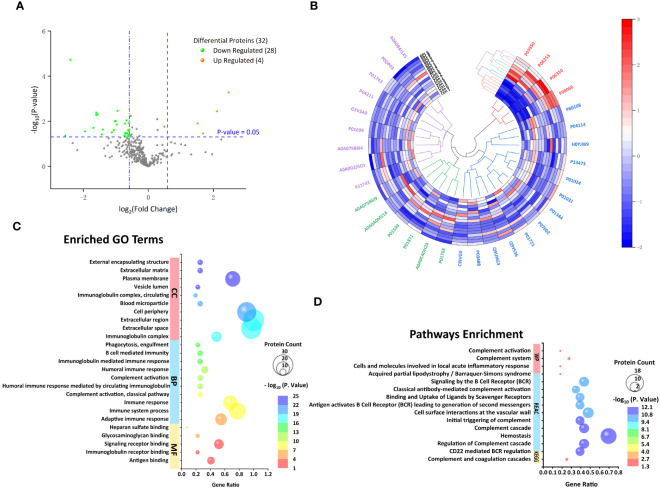
Follicular fluid proteomic and bioinformatic analysis. **(A)** Volcano plot of all proteins. Significantly upregulated proteins (Fc ≥ 1.5, *p* < 0.05) in orange and significantly downregulated proteins (Fc ≤ 0.67, *p* < 0.05) in green. **(B)** Hierarchical clustering analysis of 32 differentially expressed proteins (DEPs) between the URPL and control groups (clustered four times based on Pearson correlation). Each row in the figure represents a sample; each column is a protein. The colors represent different expression levels. **(C)** The top 10 GO terms on cellular component (CC), biological process (BP), and the only five molecular functions (MF). **(D)** The only KEGG pathway, the top 10 Reactome (REAC), and four Wiki pathways (WP).

The proteomic profile was analyzed solely in the follicular fluid samples. A total of 313 proteins were identified (**Supplementary Table 1**) in the URPL group compared to the control group. A total of 84 proteins were identified as differentially expressed based on the cutoff of the fold change (FC) ≥1.5; among these, 15 proteins were upregulated, and 69 proteins were downregulated. Comparative proteomic analysis further showed 32 proteins significantly differentially expressed with FC ≥1.5 and with *p*-values < 0.05, including four upregulated proteins and 28 downregulated proteins (**Table 3**, **Figure 2A**). To learn more about the expression correlation of the DEPs, Pearson clustering analysis was used to group the DEPs into clusters (**Figure 2B**).

**Table 3:** Differentially expressed proteins in FF identified by dDIA.

UniProt accession	Protein name	Corresponding gene	Fc	*p*-value
P03950	Angiogenin	ANG	4.55	0.001
P08253	72 kDa type IV collagenase	MMP2	4.33	0.004
P06310	Immunoglobulin kappa variable 2–30	IGKV2–30	3.21	0.035
P98066	Tumor necrosis factor-inducible gene 6 protein	TNFAIP6	2.87	0.013
P80108	Phosphatidylinositol-glycan-specific phosphoLipase D	GPLD1	0.66	0.026
P01766	Immunoglobulin heavy variable 3–13	IGHV3–13	0.66	0.012
P01871	Immunoglobulin heavy constant mu	IGHM	0.66	0.04
P21741	Midkine	MDK	0.66	0.012
P01594	Immunoglobulin kappa variable 1D-33	IGKV1D-33	0.66	0.040
P13473	Lysosome-associated membrane glycoprotein 2	LAMP2	0.66	0.046
P01024	Complement 3	C3	0.65	0.041
H0YJW9	Uncharacterized protein (Fragment)	/	0.65	0.025
P01344	Insulin-like growth factor II	IGF2	0.64	0.033
P07602	Prosaposin	PSAP	0.63	0.009
P0DP03	Immunoglobulin heavy variable 3–30	IGHV3–30	0.63	0.050
A0A0B4J1X5	Immunoglobulin heavy variable 3–74	IGHV3–74	0.62	0.036
P01031	Complement 5	C5	0.61	0.013
P04211	Immunoglobulin lambda variable 7–43	IGLV7–43	0.61	0.033
A0A0G2JSC0	Immunoglobulin lambda variable 5–45	IGLV5–45	0.60	0.043
A0A075B6I4	Immunoglobulin lambda variable 10–54	IGLV10–54	0.52	0.003
P01699	Immunoglobulin lambda variable 1–44	IGLV1–44	0.51	0.014
P01721	Immunoglobulin lambda variable 6–57	IGLV6–57	0.50	0.010
A0A0A0MS14	Immunoglobulin heavy variable 1–45	IGHV1–45	0.49	0.010
P62937	Peptidyl-prolylcis-trans isomerase A-like 4A	PPIA	0.48	0.025
A0A075B6I9	Immunoglobulin lambda variable 7–46	IGLV7–46	0.46	0.031
G3V3A0	Alpha-1-antichymotrypsin	SERPINA3	0.38	0.024
Q9UHG3	Prenylcysteine oxidase 1	PCYOX1	0.37	0.039
P00488	Coagulation factor XIII A chain	FXIIIA	0.34	0.005
P04114	Apolipoprotein B-100	APOB	0.33	0.004
A0A0C4DH24	Immunoglobulin kappa variable 6–21	IGKV6–21	0.33	0.005
C9JVG0	Serotransferrin (Fragment)	TF	0.31	0.020
P01743	Immunoglobulin heavy variable 1–46	IGHV1–46	0.26	0.028

### Gene Ontology functional annotation and Kyoto Encyclopedia of Genes and Genomes Pathway Enrichment Analysis

We investigated the functional annotation and enrichment analysis using the g:Profiler web service (http://biit.cs.ut.ee/gprofiler/). A total of 5, 60, and 32 GO terms were significantly enriched in molecular functions (MF), BP, and cellular components (CCs) (**Supplementary Table 2**; **Figure 2C**). Within the MF, observations included antigen binding, immunoglobulin receptor binding, signaling receptor binding, glycosaminoglycan binding, and heparan sulfate binding. In terms of BP, most were closely associated with immune response, complement activation, membrane invagination, phagocytosis, recognition, and activation of inflammatory cells. Consistent with BP GO term retrieval, proteins were enriched in CC such as immunoglobulin complex, extracellular space, and cell periphery. In addition, one KEGG pathway, 39 REAC pathways, and four WP were enriched (**Supplementary Table 2**; **Figure 2D**). KEGG pathways analysis revealed that the DEPs were enriched in complement and coagulation cascades. Consistently, REAC pathways analysis indicated that proteins were involved in the complement cascade, including regulation of complement cascade, initial triggering of complement, classical antibody-mediated complement activation. On the other hand, immune and inflammatory responses, hemostatic and thrombotic processes, and infections were also involved in the URPL pathway, for instance, CD22-mediated B-cell receptor (BCR) regulation, antigen activating BCR leading to generation of second messengers, cell surface interactions at the vascular wall, binding and uptake of ligands by scavenger receptors, and role of phospholipids in phagocytosis. WP enrichment analysis indicated that in addition to the complement-associated pathway, acquired partial lipodystrophy/Barraquer–Simons syndrome, along with cells and molecules involved in local acute inflammatory response, also played a vital role.

**Figure 3:**
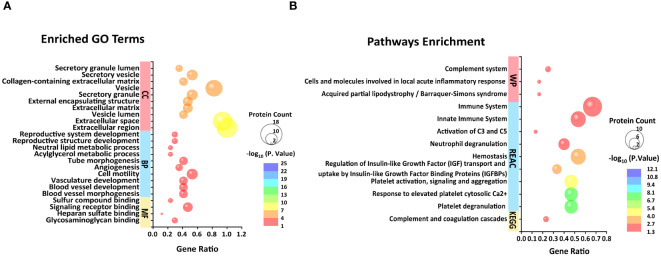
Top 10 BP, CC, and four MF **(A)** and unique KEGG pathway, top 10 REAC pathways, and three WP pathways **(B)** enriched from 16 differential proteins in FF excluding immunoglobulins and one uncharacterized protein.

Among the 32 DEPs, nearly half (14) of which were classified as immunoglobins, a singular protein was labeled as "functional unknown," annotated as "uncharacterized protein." To discover latent functions and connectivities that may exist in the rest of the 16 proteins, we performed a parallel functional annotation and enrichment analysis. More dissimilar GO terms were enriched as expected, and major changes in BP related to the regulation of angiogenesis and vascular development were observed to increase. Particularly, the enrichment in this case of GO terms related to lipid metabolism (acylglycerol metabolic process, neutral lipid metabolic process, regulation of lipid metabolic process, neutral lipid biosynthetic process, acylglycerol biosynthetic process, and organonitrogen compound metabolic process) and reproductive development (reproductive structure development, reproductive system development, developmental process involved in reproduction, female gamete generation, ovarian follicle development, and ovulation cycle) were noted (**Supplementary Table 3**; **Figure 3**). In addition, human disease enrichment analysis applying ToppGene revealed significant associations between the 16 proteins and 73 disease terms (**Supplementary Table 4**) with a Bonferroni FDR <0.05. Of these, cardiovascular system diseases were the most common. To a lesser extent, tumors, autoimmune diseases, and fetal growth retardation are also included (**Supplementary Table 4**).

### Protein–protein interaction network analysis

**Figure 4:**
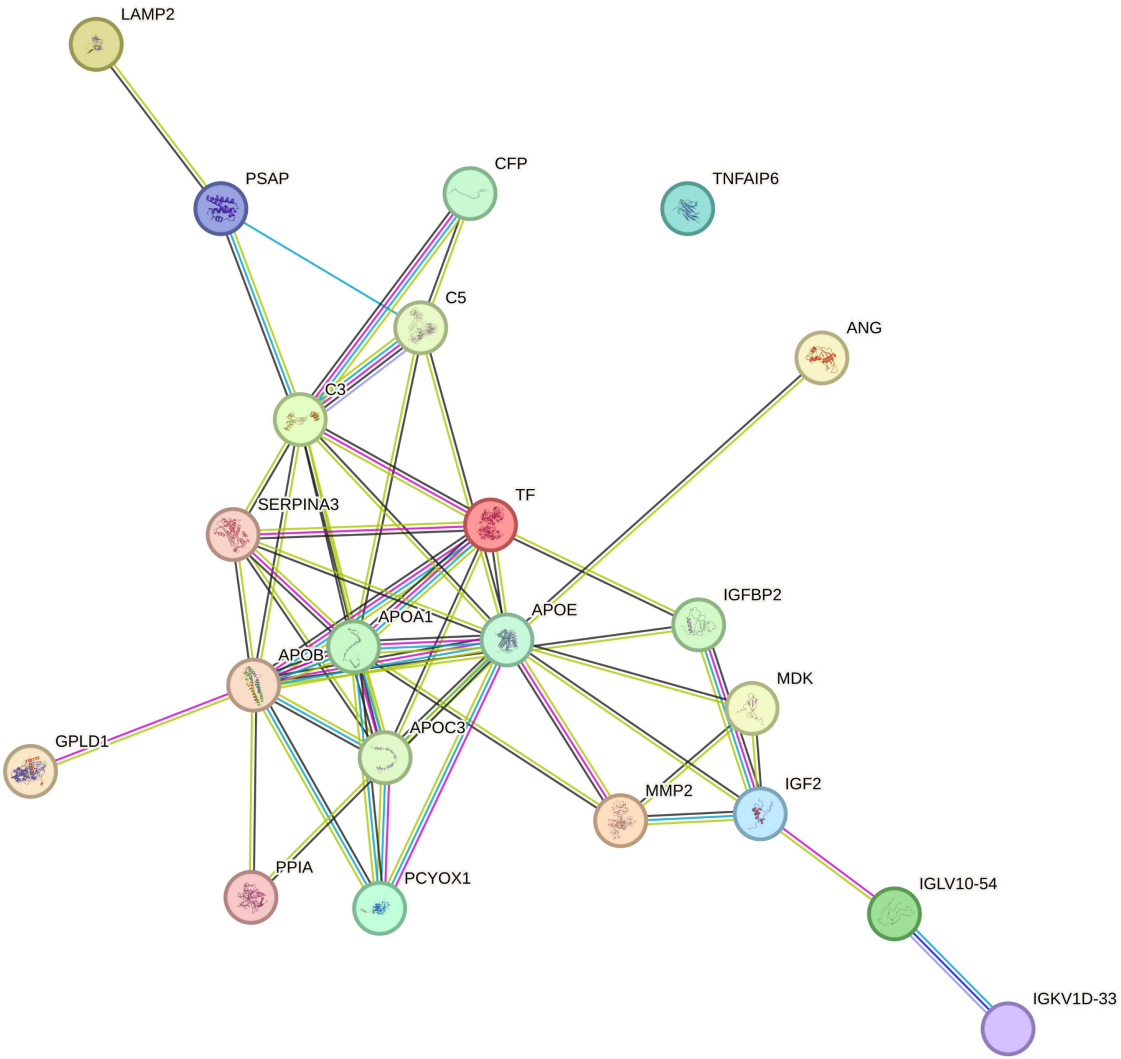
Protein–protein interaction (PPI) networks involved 22 nodes and 49 edges, as derived from the STRING database.

The PPI network of DEPs was built by using the STRING online database, which consists of 22 nodes and 49 edges in line with interaction score > 0.4 (*p* < 1.0e−16) (**Figure 4**). Subsequently, we identified the top 10 hub proteins from PPI networks based on the maximal clique centrality (MCC) method utilizing the Cytoscape plug-in Cytohubba, including APOA1, APOB, APOC3, APOE, complement 3 (C3), transferrin (TF), serpin peptidase inhibitor clade A member 3 (SERPINA3), prenylcysteine oxidase 1 (PCYOX1), complement 5 (C5), and insulin-like growth factor 2 (IGF2).

### Drug–gene interaction network

The 10 hub proteins identified from the above PPI network were explored for drug–gene interactions through the DGIdb database. A total of 65 potential therapeutic drugs for URPL were identified targeting eight proteins, excluding SERPINA3 and PCYOX1 (**Supplementary Table 5**). Currently, prednisone, vitamin E, heparin, and human growth hormone have been applied in the clinical practice of URPL.

### Expression of APOB in FF and peripheral blood

**Figure 5:**
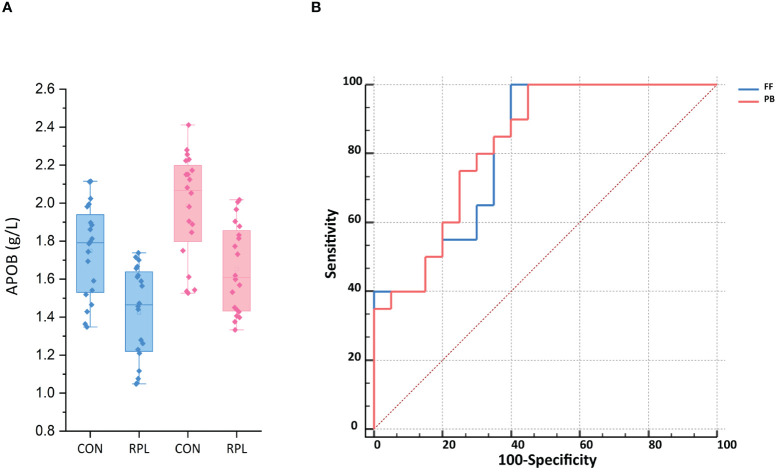
**(A)** The APOB expression in follicular fluid (FF) and peripheral blood (PB) was significantly lower in the URPL group compared to the control group (CON) by ELISA (*p* < 0.001). Statistical significance was calculated by Student's t-test. **(B)** ROC-AUC confirmed the predictive ability of APOB of FF and PB to be 81.5% (*p* < 0.01) and 82.75% (*p* < 0.05), respectively.

The ELISA test showed that the APOB level in FF for the URPL group was 1.43 ± 0.24 g/L, significantly lower than that in the control group at 1.75 ± 0.24 g/L (*p* < 0.001). Additionally, we validated APOB levels in peripheral blood (PB). Overall, the APOB concentration in PB was significantly higher than that in FF. Similarly, the level of APOB expression in PB was significantly lower in the URPL group than in the control group (1.65 ± 0.23 g/L vs. 1.99 ± 0.27 g/L, *p* < 0.001) (**Figure 5A**). The area under the receiver operating characteristic curve (ROC-AUC) was applied to assess the predictive model. The score performance yielded in FF was 81.50% [95% confidence interval (CI), 68.31%–94.69%; *p* < 0.001] and in peripheral blood was 82.75% [95% CI, 70.15%–95.35%; *p* < 0.001) (**Figure 5B**). The sensitivity for both FF and peripheral blood was 100%, and the specificity was 60% and 55%, respectively.

## Discussion

In this study, we analyzed protein profiles in FF using the dDIA proteomics approach in patients with URPL, validating and comparing them in peripheral blood and FF as potential predictive biomarkers for URPL. Overall, 32 differential proteins were identified. GO function and KEGG pathway enrichment analyses suggested that FF proteins found to be dysregulated in URPL patients were significantly enriched in the complement and coagulation cascade pathway.

Normal pregnancy maintenance is a complex and delicate process that relies on the support of a well-developed immune system. A fetus implanted in the maternal endometrium as a semi-allogeneic graft requires the immunoregulatory network at the mother–fetus interface to be tolerant and to ensure that it grows and develops harmlessly in the mother's womb. Numerous studies have confirmed that patients with URPL often exhibit more pronounced immunological imbalance *in vivo* (14, 15). Sun (16) and Cui et al. (17) separately applied TMT and iTRAQ LC-MS/MS to compare FF and serum from URPL patients with women with primary infertility and no history of miscarriage, respectively. In both studies, enrichment was observed in the immunoglobulin gamma Fc region receptor (FcγR)-mediated phagocytosis pathway, according to the KEGG analysis. Although this immune pathway was not enriched in this study, the significant up- and downregulation of immunoglobulins suggests the presence of an immune imbalance in individuals URPL.

The complement system is comprised of soluble proteins found in the blood and other bodily fluids, which are essential components of both innate and adaptive immunity (18). Initially present in an inactive form within the body, complement becomes activated by specific triggers, recognizing pathogen-associated molecular patterns (PAMPs) through a series of circulating pattern-recognition proteins (PRPs). This activation sets off a cascade of enzymatic reactions, ultimately exerting various biological effects such as immune surveillance, removal of apoptotic cells, and maintenance of cellular integrity and tissue homeostasis (19). Additionally, complement serves as a crucial inflammatory mediator, capable of inducing diverse inflammatory responses including chemotaxis, and kallikrein-like effects (20). This non-sterile inflammation triggers the release of damage-associated molecular patterns (DAMPs), such as ATP, high mobility group box 1 (HMGB1), and hyaluronic acid, which further stimulate the release of pro-inflammatory mediators from various immune cells. Consequently, this cascade leads to inflammatory cell recruitment and activation of the immune system (20). Moreover, the complement system and inflammatory molecules significantly contribute to thrombosis (21). Together, these systems form an intricate network that interacts, activates, and regulates each other to maintain immune equilibrium. The cross-talk between the complement and coagulation cascades protects against maternal rejection of the embryo and benefits the maintenance of a normal pregnancy; when these cascades are over-activated, the intrinsic feedback loops of immune activation simultaneously induce compensatory anti-inflammatory responses that rapidly amplify their otherwise targeted responses, leading to maternal–fetal interface or systemic inflammation and then to miscarriage.

In this study, 32 differentially expressed proteins were identified. Among them, angiogenin (ANG), matrix metallopeptidase-2 (MMP2), immunoglobulin kappa variable (IGKV) 2–30, and TNFAIP6 were highly expressed, while the remaining 28 proteins showed low expression levels. Apart from immunoglobulins and complement, which are directly involved in immune regulation, proteins such as C3, C5, tumor necrosis factor alpha-induced protein 6 (TNFAIP6), SERPINA3, midkine (MDK), peptidyl-prolyl cis-trans isomerasa (PPIA), prosaposin (PSAP), glycosylphosphatidylinositol-specific phospholipase D1 (GPLD1), and transferrin (TF) were identified as potent modulators of inflammatory response. Among these, SERPINA3 (22), MDK (23), PPIA (24), and GPLD1 (25) have been implicated in vascular endothelial damage and increased the risk of thrombosis by mediating inflammatory responses. The expression of ANG stimulates MMP2 expression via phosphorylation of ERK1/2 (26), leading to vascular endothelial cell damage, activation of the coagulation cascade, and induction of microvascular thrombosis. PCYOX1, a recently identified protein, is involved in thrombosis, and its absence leads to platelet hyporesponsiveness and arterial thrombosis (27). Coagulation factor XIIIA1 (FXIIIA1, a subunit of the coagulation factor FXIIIA) participates in the final stage of the coagulation cascade and is also involved in the process of angiogenesis (28). Moreover, FXIIIA is involved in alternative activation of macrophages, serving as a crucial factor linking coagulation to inflammation (29).

FF serves as the immediate natural environment for oocyte growth and development. Its constituents are derived not only from the selective diffusion of plasma but also from the secretion of ovarian granulosa cells. Within FF, a diverse array of proteins and biologically active molecules collectively form an extensive network of interconnected signaling pathways, playing crucial roles in oocyte meiosis, maturation, fertilization, and subsequent embryonic development. It has been shown that angiogenesis is an important part of follicular development (30) and that the renin–angiotensin system (RAS) is present locally in the ovary (31). As such, there has been increasing evidence in the recent years that some of the above proteins involved in the complement and coagulation cascade pathways also play important roles in ovarian and embryonic development. For example, ANG acts not only in the vascular control of ovarian function, corpus luteum formation, and luteolysis (31), but also as an autocrine/paracrine factor regulating steroidogenesis and promoting different cellular responses in the ovary (32). MMP2 in human FF increase with follicular growth, are highly expressed in the corpus luteum, and improve fertilization (33). MMP2 activity is also higher in the FF of cystic follicles than in the FF of preovulatory follicles (34). Recent evidence has shown that human oviductal epithelial cells produce complement protein 3 (C3) and its derivatives, C3b and inactivated complement 3b (iC3b) (35), which are important embryonic trophic factors that stimulate preimplantation embryo development and are involved in cell–cell interactions during fertilization and/or implantation. PSAP has also been found to be present in the epithelial cells of the fallopian tube and plays an important role in providing an optimal environment for gametes and/or spermatozoa in the ampulla during pregnancy (36), and is involved in early oogenesis. IGF2 is an important regulator of the mammalian reproductive axis, mediating steroidogenesis through activation of human luteinized granulosa cells (37). It also participates autocrinally in mitotic cell cycle activation of granulosa cells, plays a key role in follicular maturation, and stretches to all embryonic stages in the post-implantation period (38). Transferrin-Fe^3+^ accumulates in FF as follicular diameter increases; it may also minimize oxidative damage induced by exposure of cumulus oophorus cells (COCs) to endometrial ectopic fluid (39). Endometriosis-associated infertility may be due to a significant reduction in TF with iron overload resulting in oocyte immaturity (40). Recently, TNFAIP6 and MDK were found to be expressed in human cumulus cells. TNFAIP6 takes part in the meiotic resumption and expansion of cumulus and regulates ovulation via the ERK1/2 pathway. The expression of TNFAIP6 was higher in the cumulus of unfertilized oocytes than that of fertilized oocytes (41). MDK interacted with various transmembrane molecules on the cumulus granulosa cells to promote follicular growth, dominance, and differentiation, and cytoplasmic maturation of the oocyte (42). We already know that an imbalance of inflammation, microthrombosis, and immune microenvironment at the maternal–fetal interface is an important driver of spontaneous abortion and embryo loss (43). Nonetheless, in the context of our findings, these observations suggest that this disequilibrium may be present early in oogenesis and predispose it to undesirable pregnancy outcomes.

We found that APOs occupy an important part of the network when we performed PPI network and key protein analyses. APOs are essential components of lipid metabolism and transport, and their roles in cardiovascular disease are well-known, yet the understanding of their function in reproduction beyond steroidogenesis is still very limited. Li et al. (44) argued that lipid homeostasis is critical for early embryonic development, as cholesterol synthesis is required to support the membrane biosynthetic demands as cell proliferation accelerates. However, evidence suggests that pre-implantation embryos do not have the capacity to yield cholesterol and that the cholesterol required for early embryonic development is entirely dependent on its intracellular level in the post-fertilization oocyte (45, 46). As such, cholesterol is imperative for oocyte and embryo development. In contrast, it was shown that APOA1, APOB, APOE, and APOC are present in human FF in different specific cholesterol lipoprotein particles, which may be associated with age-associated decline in oocyte maturation and fertility potential (47). It was concluded that changes in APO levels in FF are likely to be a marker for the genesis of mature oocytes.

APOA1 acts as a carrier of steroid precursors and plays a key role in transferring cholesterol to granulosa cells and, in concert with paraoxonase 1 (PON1), exerts an antioxidant effect that protects oocytes from toxic damage (48). APOA1, as a major structural protein component of high-density lipoprotein (HDL), also exerts a local anti-inflammatory effect in conjunction with HDL, increasing the general protective capacity for oocyte development that enhances fertilization (49). Von Wald et al. (47) first reported the expression of APOB in human FF, and studies have confirmed the presence of APOB-containing lipoprotein receptors on oocytes, whereas granulosa cells utilize secretion of APOB-containing lipoproteins to prevent triglyceride (TG) accumulation (46). In our study, APOB expression was significantly downregulated (**Table 3**, FC = 0.33, *p* = 0.004) and is a key protein in the PPI network (**Figure 4**). Therefore, the expression of APOB in FF was measured further by ELISA, and we found that it was significantly lower in patients with URPL than in the control group (1.43 ± 0.24 g/L vs. 1.75 ± 0.24 g/L, *p* < 0.001). In order to investigate the relationship between APOB in FF and peripheral blood and its potential application as a clinical biomarker, we therefore simultaneously tested APOB levels in plasma. Encouragingly, we found that APOB expression in plasma (1.65 ± 0.23 g/L vs. 1.99 ± 0.27 g/L, *p* < 0.001) was parallel to that in the FF and was slightly lower in FF than in plasma. The ROC-AUC for URPL prediction by APOB in FF and plasma was 81.50% and 82.75%, respectively (*p* < 0.001). Scalici et al. (46) checked APOB levels in FF and found that the number of good-quality embryos was higher in the group with higher APOB (4.0 ± 1.6 g/L vs. 1.2 ± 1.3 g/L, *p* < 0.05), and the clinical pregnancy rate was significantly higher than that of the control group (69.1% vs. 23.1%; *p* < 0.05). These data reinforce our view suggesting that APOB present in FF and plasma might predict oocyte and embryo quality and onward successful implantation and pregnancy. The primary role of APOE in the ovarian tissue is to transport cholesterol into the follicle for steroidogenesis (50), whereas APOC3 is involved in oocyte growth (51). Nonetheless, proteins do not work individually but as part of macromolecular complexes. It remains unclear how lipoproteins interact with each other and with other proteins in the FF, and this needs to be fully elucidated.

Similarly, in the enrichment analysis of human diseases, cardiovascular disorders were the most common, followed by tumors, autoimmune disorders, and fetal growth retardation. This suggests that URPL may share common molecular mechanisms with cardiovascular conditions and provides an opportunity to further explore the underlying pathological mechanisms of URPL and future molecular interventions. In current clinical practice, corticosteroids, azathioprine, cyclosporine with their powerful anti-inflammatory effects, and inhibition of autoimmune reactions could reduce the production of immune complexes (52); low molecular heparin or combined with aspirin for anticoagulation could unblock the microcirculation of the villi (53); and hydroxychloroquine, an anti-malarial drug, could inhibit antigen presentation and lymphokine activation (54), with a view to alleviate the adverse outcome of pregnancy. However, the therapeutic mechanisms of these approaches presently available for URPL are concerned with the maternal–fetal interface but not yet with follicular and oocyte developmental stages. Furthermore, studies have shown that growth hormone (GH) regulates follicle-stimulating hormone (FSH) activity on granulosa cells by upregulating local IGF1 synthesis (55) and has recently been used in IVF to increase ovarian sensitivity to the effects of gonadotropins and to increase the number of mature eggs, the rate of fertilization, the number of transferrable embryos, the pregnancy rate, and the live birth rate (56). However, there is evidence that GH can be involved in lipoprotein regulation through IGF1 (57). Therefore, it remains to be investigated whether lipoproteins locally in the follicle are also involved by the same mechanism in the regulation of sex hormones and egg development by GH.

## Conclusions

URPL is a disorder characterized by dysregulation of the immune network intersecting with coagulation and inflammatory response alike with hypertension and tumors. This imbalance may not just be confined to the maternal–fetal interface, but extends as far back as the developmental stage of the egg. Therefore, early intervention therapy is necessary in women with URPL to address the underlying risk of inflammatory milieu, tissue damage, and microthrombosis. Second, the APO-centered protein regulatory network in the follicular fluid provides new candidate targets and biomarkers for URPL and fertility potential. The mechanisms of APO-mediated oocyte maturation and embryonic development are not yet fully understood, and further studies are necessary to determine the exact mechanism of action of these proteins and the potential role of their compounds in URPL and to provide prospective drug targets.

## Data availability statement

The datasets presented in this study can be found in online repositories (https://proteomecentral.proteomexchange.org) (58, 59). The names of the repository/repositories and accession number(s) can be found below: PXD051475 (ProteomeXchange).

## Ethics statement

The studies involving humans were approved by Reproductive Medicine Ethics Committee of the First Hospital of Lanzhou University (LDYYSZLL2022-05). The studies were conducted in accordance with the local legislation and institutional requirements. The participants provided their written informed consent to participate in this study.

## Author contributions

KL: Conceptualization, Formal analysis, Funding acquisition, Investigation, Methodology, Project administration, Visualization, Writing – original draft, Writing – review & editing. XX: Formal analysis, Investigation, Methodology, Writing – review & editing. LS: Investigation, Methodology, Resources, Writing – review & editing. HL: Methodology, Resources, Writing – review & editing. YJ: Resources, Writing – review & editing. XM: Resources, Writing – review & editing. BS: Supervision, Visualization, Writing – review & editing. CM: Supervision, Visualization, Writing – review & editing.

## Supplementary material

Supplementary Table 1 Proteins expressed in FF.

Supplementary Table 2 GO annotation and KEGG pathway enrichment analysis for 32 differential proteins.

Supplementary Table 3 GO annotation and KEGG pathway enrichment analysis for 16 differential proteins.

Supplementary Table 4 Disease classes annotation from ToppGene.
