# Supplementary material for: Proteomics profiling reveals lipid metabolism abnormalities during oogenesis in unexplained recurrent pregnancy loss
Source: Front Immunol. 2024 Aug 8;15:1397633. doi: 10.3389/fimmu.2024.1397633 (PMC11339622; doi:10.3389/fimmu.2024.1397633)
Supplement: Supplementary file 5 [file Table_5.docx]

Table S5 Candidate drugs targeting hub proteins

| **Hub Gene** | **Drug** | **FDA Approval** | **Drug Class** | [**Query Score**](https://dgidb.org/score) | [**Interaction Score**](https://dgidb.org/score) |
| --- | --- | --- | --- | --- | --- |
| APOE | [LUTEIN](https://dgidb.org/drugs/LUTEIN) |  |  | 4.3 | 3.64 |
|  | [GANCICLOVIR](https://dgidb.org/drugs/GANCICLOVIR) |  |  | 2.15 | 1.82 |
|  | [SOYBEAN OIL](https://dgidb.org/drugs/SOYBEAN%20OIL) |  |  | 1.72 | 1.45 |
|  | [IRBESARTAN](https://dgidb.org/drugs/IRBESARTAN) | Yes | Antihypertensive agents | 1.72 | 0.73 |
|  | [ACENOCOUMAROL](https://dgidb.org/drugs/ACENOCOUMAROL) | No | Anticoagulants | 1.43 | 1.21 |
|  | [FENOFIBRATE](https://dgidb.org/drugs/FENOFIBRATE) | Yes | Antilipemic agents | 0.89 | 0.25 |
|  | [PRAVASTATIN](https://dgidb.org/drugs/PRAVASTATIN) | Yes | Anticholesteremic agents | 0.74 | 0.31 |
|  | [ATORVASTATIN](https://dgidb.org/drugs/ATORVASTATIN) | Yes | Anticholesteremic agents | 0.72 | 0.3 |
|  | [WARFARIN](https://dgidb.org/drugs/WARFARIN) | Yes | Anticoagulants | 0.72 | 0.3 |
|  | [RITONAVIR](https://dgidb.org/drugs/RITONAVIR) |  |  | 0.68 | 0.57 |
|  | [FLUVASTATIN](https://dgidb.org/drugs/FLUVASTATIN) | Yes | Antihypecholesterolemic agent | 0.61 | 0.52 |
|  | [LORAZEPAM](https://dgidb.org/drugs/LORAZEPAM) | Yes | Anticonvulsants, hypnotics and sedatives, anti-anxiety agents | 0.39 | 0.33 |
|  | [TRIAMCINOLONE](https://dgidb.org/drugs/TRIAMCINOLONE) | Yes | Glucocorticoids | 0.37 | 0.32 |
|  | [TROGLITAZONE](https://dgidb.org/drugs/TROGLITAZONE) |  |  | 0.34 | 0.29 |
|  | [STAUROSPORINE](https://dgidb.org/drugs/STAUROSPORINE) |  |  | 0.32 | 0.27 |
|  | [PREDNISONE](https://dgidb.org/drugs/PREDNISONE) | Yes | Antiinflammatoryagent, corticosteroid | 0.27 | 0.23 |
|  | [SIMVASTATIN](https://dgidb.org/drugs/SIMVASTATIN) | Yes | Anticholesteremic agents | 0.16 | 0.13 |
| APOA1 | [CHEMBL247920](https://dgidb.org/drugs/CHEMBL247920) |  |  | 8.61 | 15.46 |
|  | [APABETALONE](https://dgidb.org/drugs/APABETALONE) | No | Antiatherosclerotic agent | 2.15 | 3.86 |
|  | [FENOFIBRATE](https://dgidb.org/drugs/FENOFIBRATE) | Yes | Antilipemic agents | 1.34 | 0.8 |
|  | [GLUCAGON](https://dgidb.org/drugs/GLUCAGON) |  |  | 0.96 | 1.72 |
|  | [FUROSEMIDE](https://dgidb.org/drugs/FUROSEMIDE) | Yes | Antihypertensive agents, diuretics | 0.78 | 1.41 |
|  | [LAMIVUDINE](https://dgidb.org/drugs/LAMIVUDINE) |  |  | 0.33 | 0.59 |
|  | [TESTOSTERONE](https://dgidb.org/drugs/TESTOSTERONE) | Yes | Hormone replacement agents | 0.24 | 0.43 |
|  | [ALCOHOL](https://dgidb.org/drugs/ALCOHOL) |  |  | 0.2 | 0.18 |
| APOB | [CHOLESTYRAMINE](https://dgidb.org/drugs/CHOLESTYRAMINE) |  |  | 2.87 | 2.29 |
|  | [MIPOMERSEN](https://dgidb.org/drugs/MIPOMERSEN) |  |  | 2.15 | 1.72 |
|  | [IRBESARTAN](https://dgidb.org/drugs/IRBESARTAN) | Yes | Antihypertensive agents | 1.72 | 0.69 |
|  | [ATORVASTATIN](https://dgidb.org/drugs/ATORVASTATIN) | Yes | Anticholesteremic agents | 1.43 | 0.57 |
|  | [FENOFIBRATE](https://dgidb.org/drugs/FENOFIBRATE) | No | Antilipemic agents | 1.34 | 0.36 |
|  | [LOMITAPIDE](https://dgidb.org/drugs/LOMITAPIDE) | No | anticholesterolaemic agent | 1.08 | 0.86 |
|  | [VITAMIN E](https://dgidb.org/drugs/VITAMIN%20E) |  |  | 0.78 | 0.62 |
|  | [PRAVASTATIN](https://dgidb.org/drugs/PRAVASTATIN) | Yes | Anticholesteremic agents | 0.49 | 0.2 |
|  | [HEPARIN](https://dgidb.org/drugs/HEPARIN) | Yes | Anticoagulants | 0.45 | 0.36 |
|  | [LOVASTATIN](https://dgidb.org/drugs/LOVASTATIN) | Yes | Anticholesterolaemic agent | 0.4 | 0.32 |
|  | [NEVIRAPINE](https://dgidb.org/drugs/NEVIRAPINE) |  |  | 0.37 | 0.3 |
|  | [TRIFLUOPERAZINE](https://dgidb.org/drugs/TRIFLUOPERAZINE) | Yes | Antipsychotic agents | 0.36 | 0.29 |
|  | [EPIGALOCATECHIN GALLATE](https://dgidb.org/drugs/EPIGALOCATECHIN%20GALLATE) |  |  | 0.33 | 0.26 |
|  | [WARFARIN](https://dgidb.org/drugs/WARFARIN) | Yes | Anticoagulants | 0.24 | 0.1 |
|  | [HYDROCORTISONE](https://dgidb.org/drugs/HYDROCORTISONE) |  |  | 0.23 | 0.19 |
|  | [ALCOHOL](https://dgidb.org/drugs/ALCOHOL) |  |  | 0.2 | 0.08 |
|  | [QUERCETIN](https://dgidb.org/drugs/QUERCETIN) |  |  | 0.11 | 0.09 |
|  | [DEXAMETHASONE](https://dgidb.org/drugs/DEXAMETHASONE) | Yes | Antiinflammatory agent,glucocorticoid | 0.1 | 0.08 |
| C3 | [COMPSTATIN](https://dgidb.org/drugs/COMPSTATIN) |  |  | 8.61 | 41.22 |
|  | [PEGCETACOPLAN](https://dgidb.org/drugs/PEGCETACOPLAN) |  |  | 4.3 | 20.61 |
|  | [CLOZAPINE](https://dgidb.org/drugs/CLOZAPINE) | Yes | Antipsychotic agents | 0.15 | 0.7 |
| TF | [ADEMETIONINE](https://dgidb.org/drugs/ADEMETIONINE) |  |  | 2.15 | 15.46 |
|  | [ADALIMUMAB](https://dgidb.org/drugs/ADALIMUMAB) | Yes | Antirheumatic agents | 0.51 | 3.64 |
| C5 | [ECULIZUMAB](https://dgidb.org/drugs/ECULIZUMAB) | Yes | Immunomodulatory agents | 21.52 | 44.16 |
|  | [TESIDOLUMAB](https://dgidb.org/drugs/TESIDOLUMAB) |  |  | 8.61 | 17.67 |
|  | [AVACINCAPTAD PEGOL SODIUM](https://dgidb.org/drugs/AVACINCAPTAD%20PEGOL%20SODIUM) | No | aptamer | 8.61 | 17.67 |
|  | [PEXELIZUMAB](https://dgidb.org/drugs/PEXELIZUMAB) |  |  | 4.3 | 8.83 |
|  | [RAVULIZUMAB](https://dgidb.org/drugs/RAVULIZUMAB) |  |  | 4.3 | 8.83 |
|  | [ZILUCOPLAN](https://dgidb.org/drugs/ZILUCOPLAN) |  |  | 4.3 | 8.83 |
|  | [NOMACOPAN](https://dgidb.org/drugs/NOMACOPAN) |  |  | 4.3 | 8.83 |
| IGF2 | [GANITUMAB](https://dgidb.org/drugs/GANITUMAB) | Yes | Antineoplastic agent | 6.46 | 6.62 |
|  | [DUSIGITUMAB](https://dgidb.org/drugs/DUSIGITUMAB) |  |  | 4.3 | 4.42 |
|  | [HUMAN GROWTH HORMONE](https://dgidb.org/drugs/HUMAN%20GROWTH%20HORMONE) |  |  | 2.87 | 2.94 |
|  | [XENTUZUMAB](https://dgidb.org/drugs/XENTUZUMAB) |  |  | 2.15 | 2.21 |
|  | [GLUTAMINE](https://dgidb.org/drugs/GLUTAMINE) |  |  | 1.43 | 1.47 |
|  | [LINSITINIB](https://dgidb.org/drugs/LINSITINIB) | No | Kinase Inhibitors | 1.23 | 1.26 |
|  | [BETAMETHASONE](https://dgidb.org/drugs/BETAMETHASONE) | Yes | Antiinflammatory agent,glucocorticoid | 0.96 | 0.98 |
|  | [ANASTROZOLE](https://dgidb.org/drugs/ANASTROZOLE) | Yes | Antineoplastic agents | 0.66 | 0.68 |
|  | [CABAZITAXEL](https://dgidb.org/drugs/CABAZITAXEL) | Yes | Antineoplastic agent | 0.51 | 0.52 |
|  | [FLUOXETINE](https://dgidb.org/drugs/FLUOXETINE) | Yes | Antidepressive agents, second-generation | 0.32 | 0.33 |
|  | [ETOPOSIDE](https://dgidb.org/drugs/ETOPOSIDE) | Yes | Antineoplastic agents | 0.17 | 0.17 |
|  | [GEFITINIB](https://dgidb.org/drugs/GEFITINIB) | Yes | Antineoplastic agents, protein kinase inhibitors | 0.13 | 0.13 |
|  | [DOCETAXEL](https://dgidb.org/drugs/DOCETAXEL) | Yes | Antineoplastic agents | 0.11 | 0.11 |
|  | [GEMCITABINE](https://dgidb.org/drugs/GEMCITABINE) | Yes | Antineoplastic agent | 0.11 | 0.11 |
